# The efficacy of “TiRobot”orthopaedic robot-assisted VS conventional fluoroscopic percutaneous screw fixation of the sacroiliac joint

**DOI:** 10.1007/s00264-022-05655-z

**Published:** 2022-12-27

**Authors:** Ningtao Li, Zongdong Zhu, Chengwei Xiao, Dan Wei, Fei Wang, Wei Zhang, Jiang Hu

**Affiliations:** 1 Sports Medicine and Health Collegee, Chengdu Sport University, Sichuan Province, No.2 Tiyuan Road, Chengdu City, 610041 China; 2 Orthopaedic Department, Sichuan Provincial People’s Hospital, 32# W. Sec 2, 1St Ring Rd, Qingyang District, Chengdu, 610072 China

**Keywords:** Sacroiliac joint screw, Injury of posterior pelvic ring, Percutaneous, TiRobot assisted, Conventional fluoroscopy assisted

## Abstract

**Purpose:**

This study is to compare the precision and safety of the orthopaedic robot with conventional fluoroscopy for assisted percutaneous sacroiliac joint screw implantation.

**Methods:**

Retrospective analysis was performed on the clinical data of 57 patients with unstable posterior pelvic ring injuries who were admitted and met the criteria between January 2017 and January 2022. All of these patients underwent percutaneous sacroiliac joint screw implantation, and their clinical data were split into two groups based on the surgical technique: a RA group (robot-assisted implantation, 30 patients, 54 screws) and a CF group (conventional fluoroscopic freehand implantation, 27 patients, 42 screws). There were 96 screws placed in total. The durations of the two groups’ operations, fluoroscopy examinations, fluoroscopy doses, total number of fluoroscopies, and intra-operative guide pin applications were noted and compared. On post-operative CT scans, the placement of each screw was assessed using the Gertzbein-Robbins classification. Finally, imaging Matta criteria were used to assess the sacroiliac joint fracture reduction. The Majeed functional score was used to assess clinical function.

**Results:**

Both groups successfully completed 57 procedures in total. In both groups, there were no consequences from vascular injury, wound infection, or urinary tract infection. Additionally, there were no complications from robotic-induced nerve injury, operating time, fluoroscopic dose, and the frequency of fluoroscopic; the number of percutaneous punctures in the RA group was lower than that of the CF group.There were statistically significant differences between the aforementioned data (*P* < 0.05). The modified Matta evaluated the effectiveness of fracture reduction. In the RA group, there was no statistically significant difference between the CF group (*P* > 0.05). According to the modified Gertzbein-Robbins classification criteria, the 54 screws implanted in the RA group were classified as follows: class A (45), class B (5), class C (4), and class D (0); the accuracy rate of the implants was 92.59%. Forty-two screws implanted in the CF group, 30 screws were defined class A, class B (3), class C (7), and class D (2). The accuracy rate of the implants was 78.57%(χ2 = 3.967, *P* < 0.05). There was a statistically significant difference between the two groups. The Majeed score 30 patients in RA group, one month post-operation, 16 considered exceptional, eight decent, six moderate, and zero bad. Post-operation more than six months,25 recorded exceptional, five decent. By the time,27 patients in CF group,12 exceptional grade, eight decent, six moderate, and one bad,one month post-operation. Post-operation more than six months,22 recorded exceptional, five decent.Both group (P > 0.05).

**Conclusion:**

“TiRobot” robot-assisted screw implant treatment for unstable posterior pelvic ring injury has a greater success rate than traditional surgery as compared to conventional percutaneous screw implant. It is a precise, secure, and minimally invasive surgical technique that can also be applied to severe pelvic injuries even congenital sacral deformities.

## Introduction

Pelvic fractures caused by high-energy trauma are usually unstable and have a high mortality rate, ranging from 10 to 16%. Severe infection can complicate early haemorrhage or late multiorgan failure [1]. The sacrococcygeal complex, which includes the bilateral ilium, sacrum, sacrococcygeal joints, and surrounding ligaments, accounts for 60% of stability. Injuries to the unstable posterior pelvic ring present orthopaedic surgeons with a therapeutic challenge. Strong fixation is an important treatment goal to reduce bleeding, facilitate recovery, and avoid long-term complications [2]. Secure fixation of the sacroiliac joint must be accomplished in order to regain stability [3]. Since 1973, percutaneous screw fixation has been regarded as the “gold standard” for the treatment of posterior pelvic ring fractures. However, sacroiliac screw fixation is the only minimally invasive method available to support the posterior pelvic ring. Less surgical trauma, less bleeding, fewer problems, a decreased likelihood of infections, and a quicker post-operative recovery are all benefits [4, 5]. There is still a significant risk of medical injury to the lumbosacral nerve roots, superior gluteal artery, and iliac artery due to the complex vascular and neurological structures of the sacroiliac joint and sacrum, ambiguous anatomical landmarks, and unclear intra-operative fluoroscopic visualization [6].

The wrong placement of the kerf or screw during implantation may be the root of these issues. Positioning mistakes are still said to occur at a rate of roughly 5%. Furthermore, traditional manual screw placement has drawbacks such as frequent X-ray fluoroscopy and a lengthy procedure time [7]. Implantation of IS screws using X-ray fluoroscopy necessitates extensive clinical experience, and the procedure still has a steep learning curve. Several studies have shown that the misplacement rate of IS screw position under conventional fluoroscopic guidance is 2–15% and the nerve injury rate is 1–7% [8].

Robot Surgical Robot Navigation and Positioning System is the third generation of surgical robot developed by Beijing TINAVI Medical Technology Company, which is the most recent generation of orthopaedic surgical robot system developed independently in China and internationally recognized and has been certified by China Food and Drug Administration (CFDA). The system adopts modular, miniaturized and universal design and achieves positioning, surgical planning, and motion navigation through spatial mapping. Due to its minimally invasive, precise, intelligent, and stable features, its indications have been extended to spinal surgery, joints, and trauma [9–13]. We have been using it to the management of pelvic fractures since January 2017. In order to compare patients receiving conventional fluoroscopic surgery with this undergoing robot-assisted screw implantation for unstable posterior pelvic ring injuries, we gathered medical data on both groups of patients.

## Methods and materials

### Patient selection

Inclusion criteria are as follows: ① pelvic fracture confirmed by X-ray and CT; ② closed unstable posterior pelvic ring fracture (Tile type B and Tile type C); ③ age >  = 18 years; ④ fresh fracture, ≤ 21 d after injury; and ⑤ no other injuries to the affected lower limb.

Exclusion criteria are as follows: ① age < 18 years; ②old fracture; ③combined with severe abdominal injury, spinal cord injury or major organ failure; ④ other injuries affecting the function of the affected lower limb; ⑤ uncooperative treatment or psychiatric illness; and ⑥ pathological fracture or patients with severe osteoporosis.

According to the surgical method, the clinical data of 57 patients with unstable posterior pelvic ring injuries who met the criteria were divided into RA group (robot-assisted implantation, 30 patients, 54 screws) and CF group (conventional fluoroscopic freehand implantation, 27 patients, 42 screws). There were 96 screws implanted in total. Every patients had informed consent.

### Clinical data

The Robot assisted (RA) group consisted of 21 men and 9 women, with an average age of 62.5 years (range from 43 to 72 years). The conventional fluoroscopic (CF) group consisted of 16 men and 11 women, ranging in age from 35 to 70, with an average age of 53.77. According to Tile, 12 cases incidences of type B and 18 cases of type C of injury to the posterior ring of the pelvis were found in the RA group. There were 17 sacroiliac joint separation instances (12 unilateral, 5 bilateral), and one sacral fracture case. There were 14 type B cases, 13 type C cases, 17 cases of sacroiliac joint separation (unilateral 12 cases, bilateral 5 cases), and two cases of sacral fracture in the conventional fluoroscopic (CF) group. None of the patients listed above displayed obvious signs of nerve damage. There was no statistically significant difference in gender, age, BIM value, trauma history, fracture classification, or average number of screws per case (*P* > 0.05). For more information, see Table 1.

**Table 1:** Patient demographics

Index	TiRobot-assisted group (*n* = 30)	Conventional fluoroscopic group (*n* = 27)	*T* value	*P* value
Age (year, x̅ ± s)	57.33 ± 9.211	53.78 ± 7.470	1.607	0.118
Gender (man/woman)	21/9	16/11	0.720	0.396
BMI	23.683 ± 3.1003	24.126 ± 3.0584	0.542	0.590
Tile typing B/C	12/18	14/13	0.805	0.370

### Treatment method

Both groups were operated on by two trauma chief surgeons.

#### CF group

The surgical position and anaesthesia skin incision was about 1 cm below the posterior superior iliac spine and about 5 cm laterally. The patient was given general anaesthesia and positioned supine on a traction bed. Take C-arm fluoroscopy; to confirm the fracture was repositioned by traction bed or circular pelvic retractor, a small incision approximately 2 cm long was made at the marked point. Before inserting the guide needle, the fascia and muscle were separated and tilted 30° to 50° forward. The sacral vertebrae were slowly passed over the sacral foramen and 1/3 of the sacrum into the sacral vertebrae through the iliac and sacral joints. After fluoroscopy of the guide needle, the correct position of the sacral screw is determined. Manually make the hollow screw along the guide needle, and make sure the position is good by taking X-ray, and then flush and suture.

#### RA group: the “TiRobot” system (TINAVI Medical Technologies, Beijing, China)

**Fig. 1:**
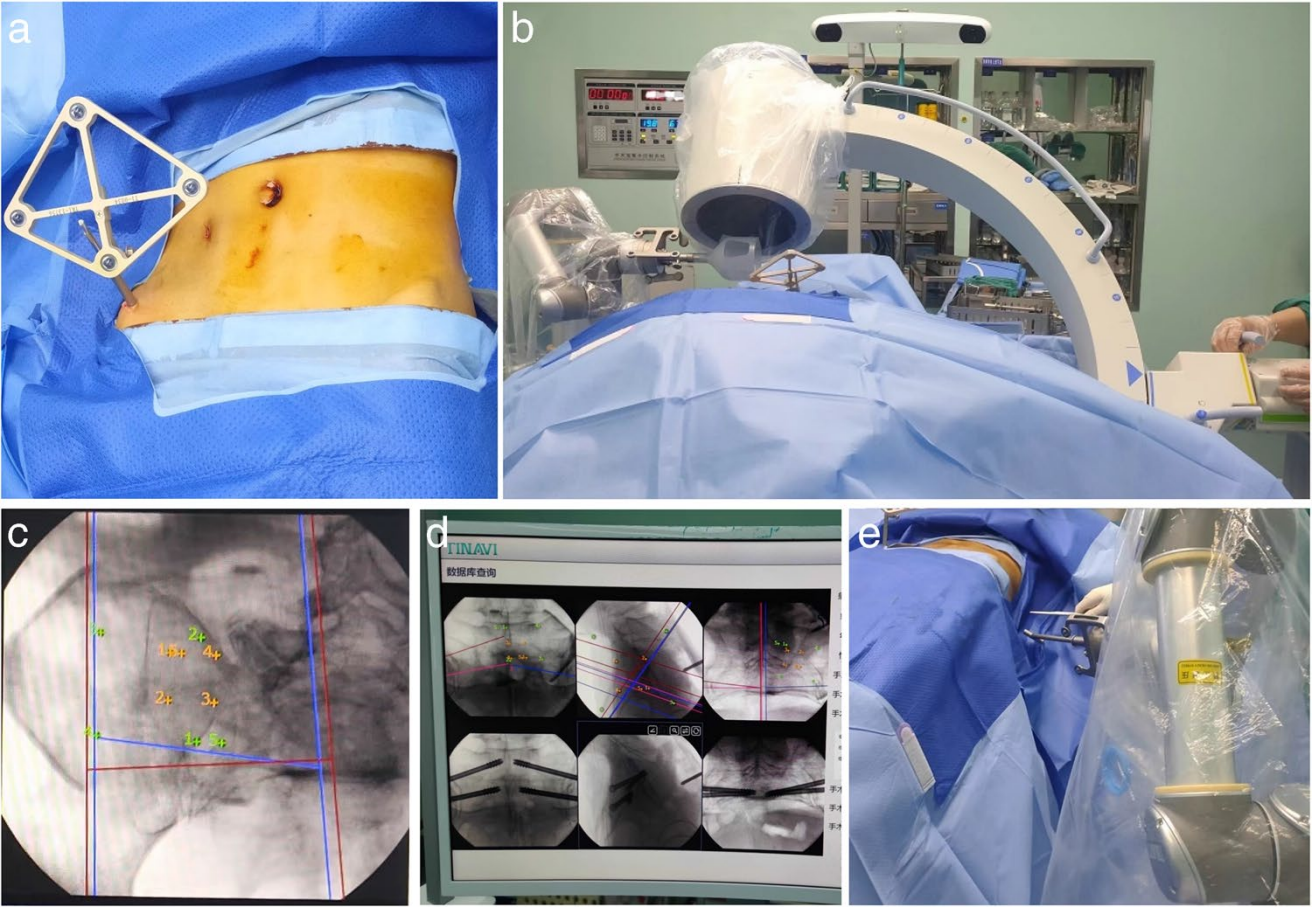
**a** A tracer is placed on the patient's anterior superior iliac spine by the surgeon. **b** Confirming all marks on the tool tracker by X-ray. **c** The entry point, angle, and length of screw were designed and locate the screw’s entry point and direction. **d** The computer plans the position of the screw. **e** The operator inserts the guide wire into the guide sleeve

A robotic arm, an optical tracking system, and an operable planning and control workstation comprise this system. Siemens’ ARCADISOrbic 3D system was used for the C-arm machine and enables the robot in real time to perform the placement of the screw. A Slovis/Ogia fixation system was used for internal fixation. All of the internal fixation materials were hollow screws, which have good histocompatibility and strength [14]. All procedures were carried out by the same surgical team. The patient was placed supine on a traction bed after a successful general anaesthesia. The surgical site was disinfected and toweled, and the fracture was repositioned with a traction bed or a circular pelvic retractor, with good repositioning visible on C-arm fluoroscopy. The contralateral anterior superior iliac spine was visualized, and a small incision was made, and a tracer was placed on the anterior superior iliac spine. The mechanical arm was fitted with a sterile C-arm sleeve, and an optical tracer and computer were attached. The C-arm X-ray machine was then used to fluoroscopically visualize the pelvic entry and exit positions, as well as to ensure that all ten markers on the positioning device were visible. The computer operating system confirmed the position and sequence of all markers. Based on the imported data, the robotic arm performed surgical screw placement planning to design the screw point, angle, and length of the percutaneous sacral screws. After replacing the positioning device with the guide sleeve and confirming the correct orientation, the “Move” button on the computer operating system’s operating interface was pressed, and the robotic arm moved the guide barrel to the surface 3 cm from the skin, the entry point, and angle of the percutaneous sacral screw, as planned. An incision of approximately 1–2 cm in length was made in the skin area, the fascia and muscle were separated, and a kerfing needle was inserted to the reserved length along the guide sleeve; the sacral screw was inserted along the guide needle, the kerfing needle was withdrawn, and the wound was irrigated before suturing. The screw position was determined using a C-arm x-ray machine (see Fig. 1).

### Data collection

The operation time, fluoroscopy time, fluoroscopy dose, fluoroscopy frequency, and frequency of guide needle use during the operation were all recorded in both groups. The fluoroscopic dose and fluoroscopic time for both groups were read directly from the main screen of the C-arm X-ray machine, where the fluoroscopic dose was the sum of the fluoroscopic dose of the continuous scan (mean 58.32 c Gy cm2) and the single fluoroscopic dose at registration.

Within 48 h post-operation, each patient has completed the X-ray and CT scan examination. The modified Matta criteria were applied to the imaging analysis of the sacroiliac joint fracture reduction; a fracture displacement of less than 5 mm was deemed excellent, 5–10 mm good, 10–20 mm acceptable, and more than 20 mm unsatisfactory. The accuracy rate of screw implantation was evaluated according the modified method of the Gertzbein-Robbins classification criteria: Class A (the screw in the central axis of sacroiliac channel, following the original design route); Class B (deviated from the central axis but did not break through the bone cortex); Class C (cortical penetration < 6 mm, the screw threaded partially cortical penetration and did not damage the adjacent blood vessels or nerves); and Class D (cortical penetration > 6 mm). Accuracy of screw placement = (number of screws of class A + number of screws of class B)/total number of implanted screws × 100%.

In order to score a total of five aspects—pain (30 points), standing (20 points), sitting (10 points), sexuality (4 points), and ability to work (36 points)—clinical function was assessed by the Majeed pelvic fracture grading method. A score of 85–100 was considered exceptional, 70–84 decent, 55–69 moderate, and below 55 bad [15]. The first time was one month post-operation, and the last follow-up was more than six months.

### Statistical analysis

For the analysis, SPSS 22.0 statistical software was employed. The measurement data were reported as mean ± standard deviation, and an independent sample *t*-test was used to compare the groups. χ^2^test was used for comparison between groups for counting data; test level α = 0.05.

## Results

Both groups successfully completed 57 procedures in total. In both groups, there were no consequences from vascular injury, wound infection, or urinary tract infection. Additionally, there were no complications from robotic-induced nerve injury. During the implementation, one lumbosacral transitional vertebrae patient in RA group, part of one screw was out of the original design path, and we corrected the direction in time. In post-operation CT scan, we find the S1 corridor’s uneven bone density of the sacroiliac channel due to the abnormal development of the sacrum, so we chose the S2 segment as pathways for iliosacral screw.

The RA group’s operating time (34.503 ± 4.767 min) was considerably less than that of the CF group (74.707 ± 2.459 min).The RA group’s fluoroscopic dose (197.995 ± 53.155cGycm^2^) was significantly lower than the CF group’s patients’ (247.355 ± 32.314cGycm^2^) X-ray exposure. The frequency of fluoroscopic in the RA group was (13.87 ± 2.688 times) and in the CF group was (35.48 ± 5.618 times). The number of percutaneous punctures in the RA group (4.93 ± 1.78 times) was lower than that of the CF group (14.48 ± 7.046 times). There were statistically significant differences between the aforementioned data (*P* < 0.05), as seen in Table 2.

**Table 2:** Comparison of surgical clinical data

Index	TiRobot-assisted group (*n* = 30)	Conventional fluoroscopic group (*n* = 27)	Test statistic	*P* value
Operation time (min, x ± s)	34.503 ± 4.7677	74.707 ± 2.4598	40.575	0.000
Patient X-ray exposure (cGy cm^2^, x ± s)	197.995 ± 53.155	247.355 ± 32.314	4.282	0.000
X-ray frequency (x ± s)	13.87 ± 2.688	35.48 ± 5.618	18.204	0.000
Puncture frequency (x ± s)	4.93 ± 1.78	14.48 ± 7.046	6.848	0.000

Using the modified Matta evaluated the effectiveness of fracture reduction. In the RA group, 22 were deemed excellent and eight as good. In the CF group, 16 got excellent point, eight as good, and three deemed acceptable. There was no statistically significant difference between the two groups, as shown in Table 3.

**Table 3:** Comparison of fracture reduction clinical data

Index	TiRobot-assisted group (*n* = 30)	Conventional fluoroscopic group (*n* = 27)	*T* value	*P* value
Matta points	22/8/0/0	16/8/3/0	3.421	0.184

According to the modified Gertzbein-Robbins classification criteria, the 54 screws implanted in the RA group were classified as follows: class A (45), class B (5), class C (4), and class D (0); the accuracy rate of the implants was 92.59%. Forty-two screws implanted in the CF group, 30 screws were defined class A, class B (3), class C (7), and class D (2). The accuracy rate of the implants was 78.57% (χ2 = 3.967, *P* < 0.05). There was a statistically significant difference between the two groups, as seen in Table 4.

**Table 4:** Accuracy rate evaluation of screw implantation clinical data

Index	TiRobot-assisted 54 screws	conventional fluoroscopic 42 screws	Test statistic	*P* value
Gertzbein-Robbins scale(A/B/C/)	45/5/4/0	30/3/7/2	3.967	0.046

The Majeed score 30 patients in RA group, one month postoperation,16 considered exceptional, eight decent, six moderate, and zero bad. Post-operation more than six months,25 recorded exceptional, five decent. By the time,27 patients in CF group,12 exceptional grade, eight decent, six moderate, and one bad,one month post-operation. Post-operation more than six months,22 recorded exceptional, five decent.Both group (P > 0.05),Table 5 for details.

**Table 5:** Comparison of functional efficacy between the two groups

Index	TiRobot-assisted group (*n* = 30)	conventional fluoroscopic group (*n* = 27)	Test statistic	*P* value
Majeed points after 1 month	16/8/6/0	12/8/6/1	1.423	0.828
Majeed points last follow-up	25/5/0/0	22/5/0/0	-	1.000

**Fig. 2:**
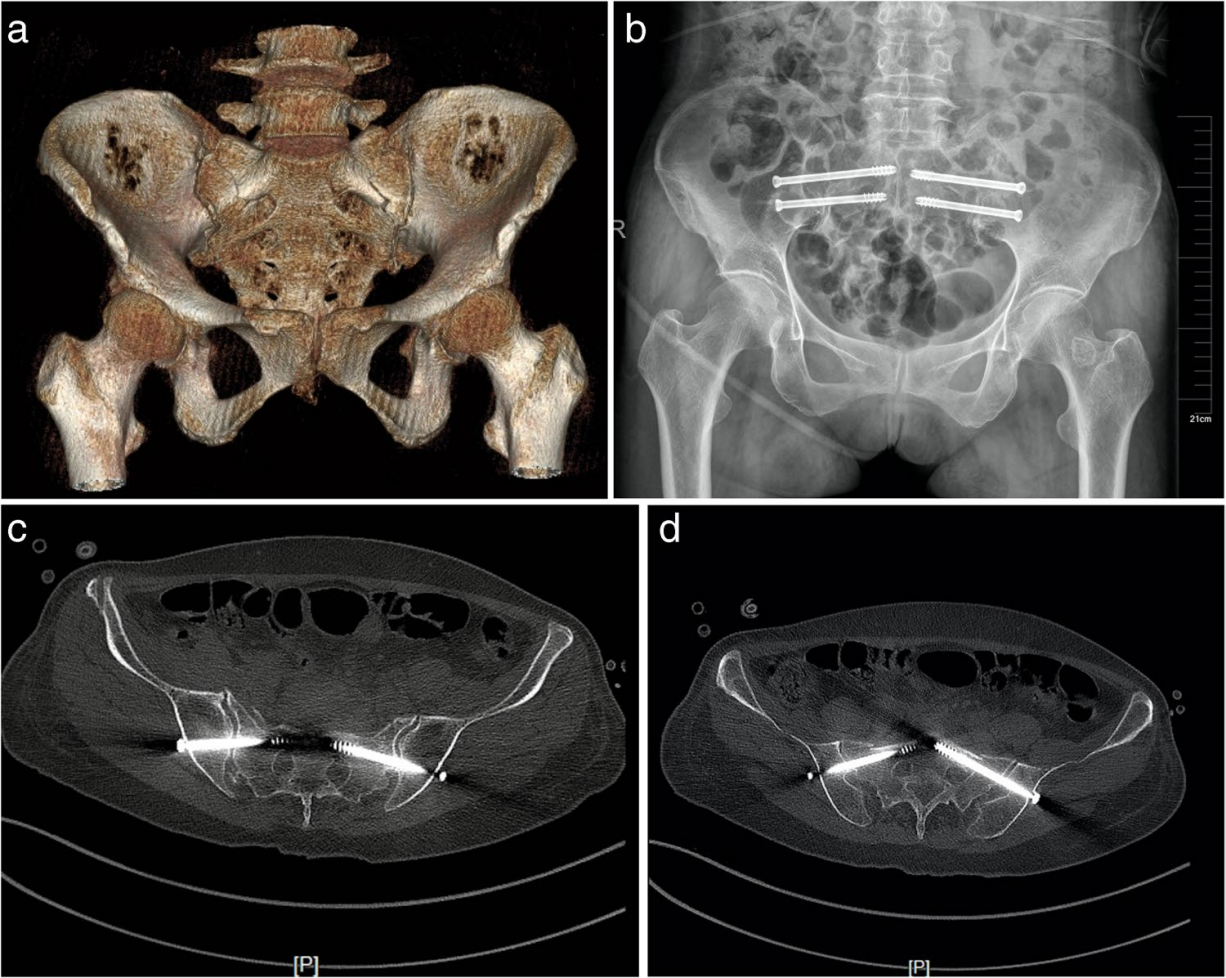
a 67-year-old female patient with bilateral sacroiliac joint injury; **a** CT scan pre-operation; **b** X-ray in post-operation; **c** post-operation CT scan showed that the S1 segment screws were well fixed; **d** post-operation CT scan indicates accurate implantation of S2 segment screw

Typical cases are shown in Fig. 2.

## Discussion

Anatomical repositioning, correction of pelvic asymmetry, and early rehabilitation are the objectives of surgical treatment for unstable Tile B2 and C pelvic ring injuries. For complex pelvic injuries, combined anterior and posterior ring (APR) fixation is used to improve overall pelvic stability. However, based on various studies, sacroiliac joint screw fixation alone for Tile B2 and C pelvic ring lesions met the same therapeutic objectives with positive outcomes, particularly for vertically unstable pelvic ring injuries. Operative time, haemorrhage, and infection risk were all reduced as compared to open reduction internal fixation treatment [16]. The risk of screw implant deviation is relatively high while doing traditional manual percutaneous pedicle screw implant surgery because it is challenging to properly control the angle of screw insertion. Percutaneous pedicle screw screwing is now easier and more accurate thanks to the orthopaedic robot’s addition of a robotic arm to the navigation system [17].

The TiRobot group’s operating time, incision length, hemorrhage, and anesthetic time were all dramatically reduced compared to the conventional groups. This was closely tied to TiRobot’s surgical strategy. The error rate of percutaneous screw implantation assisted by a “TiRobot” orthopaedic robot is 1.4% to 2.3%, according to some institutions [18]. Except for the dislocation caused by the quality of the guide needle, the loosening of the instrument arm, and the inability to maintain fracture reduction during screw placement, the authors believe that the robot cannot fully restore the shape of the sacrum and cannot recognize some abnormal sacral development. At the moment, the robot cannot reconstruct the screw channel, and screw path planning is still subject to the operator’s subjective judgment, such as the patient’s sacroiliac screw channel deformity. It is difficult to avoid peripheral neurovascular injury depending on the original computer plane.

This stage of research has some limitations. The author believes that the following points should be addressed during treatment:

1. During this statistic, the number of cases included in the study was small, but in actual clinical practice, there are still a large number of patients with concomitant sacral fractures with Denis type II and III fractures, which have a longer post-operative nerve injury recovery period and require higher intra-operative requirements for fracture repositioning, screw access selection, and screw placement accuracy. Maintaining the stability of fracture reduction is an important thing during the process of placing screws, to achieve this must by reliable equipment. More clinical experience is required to deal with patients with severe injuries or sacral variants in whom conventional screw access fixation cannot be performed.
2. Instrument and sterile area protection: The C-arm sleeve is used to shield the TiRobot and C-arm. To enable reliable identification by the optical tracking camera, the tracer is attached and secured. The protection of the ground support system needs to be activated in order to avoid displacement once the TiRobot has been placed in the desired position. Image faults and positioning accuracy can both be impacted by minor positional changes.
3. Pre-operatively, sacral variants must be ruled out and a safe access for the screw implant must be planned. Sacral deformities are estimated to affect more than 20% of adults [18, 19]. The fusion of L5 with the first sacrum is the most common cause, but it is so weakly, with limited screw access bone and poor shear and rotation resistance with a single sacroiliac joint screw for internal fixation [20]. Placing horizontal screws directly across a deformed sacrum can result in nerve injury. Screw placement feasibility can be predicted using anterior margin height and S1S2 angle [21, 22]. Earlier research on the size and orientation of the sacral fixation pathway concentrated on the S1 and S2 segments. Normal and deformed sacrums have different cross-sectional areas, and if a patient has lumbar sacralized fusion instability, the S2 or S3 as an alternative segment recommend for deformity fixation [23]. Otherwise, the lumbosacral nerve trunk, cauda equina root, and first sacral nerve are at risk of injury [24].
4. There is still a learning curve in robot operation, and screw placement path planning has not been completed in advance. It is not advised that junior doctors rely solely on existing robot technology to complete surgery. The screw placement process requires a certain amount of working experience to be carried out manually. The screwing process must be done manually by skilled workers. Otherwise, the screws are prone to slip, resulting in the fracture not being repositioned and the direction deviated.

## Conclusion

To summarize, TiRobot-assisted sacral screw placement is minimally invasive, safe, convenient, and accurate for posterior pelvic ring fractures. In comparison to freehand screw placement, robot assistance reduces the secondary injury caused by needle path deviation, achieves higher accuracy and safety than the conventional method, and reduces the number of fluoroscopy and radiation exposure during surgery. In the future, we will investigate the robot’s intelligent recognition of sacral variation, computer reconstruction of screw channel, and more intuitive provision of sacral multi-segment safe and feasible screw channel data to operators. Many scholars’ efforts are expected to maximize the advantages of precision and efficiency of orthopaedic robots and accelerate the development of clinical digitization [25].

## Data Availability

The datasets used and/or analyzed during the current study are available from the corresponding author on reasonable request.
